# Associations of Mechanistically Partitioned Type 2 Diabetes Polygenic Risk With Psychiatric and Related Phenotypes in UK Biobank

**DOI:** 10.1016/j.bpsgos.2026.100817

**Published:** 2026-08-28

**Authors:** Etsubdink T. Gebretsadik, Alisha Aman, Donald M. Lyall, Jessica Tyrrell, Madeleine M.E. Hayman, Nina Fajs, Laura M. Lyall, Rona J. Strawbridge

**Affiliations:** a School of Health and Wellbeing, University of Glasgow, Glasgow, United Kingdom; b College of Medical, Veterinary, and Life Sciences, Graduate School, University of Glasgow, Glasgow, United Kingdom; c Department of Clinical and Biomedical Sciences, Faculty of Health and Life Sciences, University of Exeter, Exeter, United Kingdom; d School of Cardiovascular and Metabolic Health, University of Glasgow, Glasgow, United Kingdom; e Deanery of Molecular, Genetic and Population Health Sciences, University of Edinburgh, Edinburgh, United Kingdom; f Centre for Clinical Brain Sciences, University of Edinburgh, Edinburgh, United Kingdom; g Health Data Research UK, Glasgow, United Kingdom

**Keywords:** Anxiety, Chronic pain, Genetics, Mood instability, Neuroticism, Type 2 diabetes

## Abstract

**Background:**

Type 2 diabetes (T2D) and psychiatric disorders frequently co-occur; however, it remains unclear whether this overlap is generalized or defined by particular T2D mechanisms and psychiatric phenotypes. We investigated whether genetically defined T2D pathways show distinct associations with psychiatric and related phenotypes.

**Methods:**

We calculated polygenic risk scores (PRSs) for 8 mechanistic T2D clusters and tested associations with psychiatric and sleep phenotypes and chronic pain in the UK Biobank. The study included 408,063 White British participants (54.1% female) and 50,214 non-overlapping White European participants (56.2% female). Models incorporated demographic, socioeconomic, lifestyle, anthropometric, and medication factors, with false discovery rate (FDR) correction across all tests.

**Results:**

Adiposity PRS showed the broadest associations. Body-fat distribution PRS was associated with atypical major depressive disorder across all models, whereas obesity-mediated insulin-resistance PRS was associated only in the minimally adjusted model. Neither PRS was associated with nonatypical depression after FDR correction. Body-fat PRS was associated with generalized anxiety disorder, mood instability, long sleep duration, and chronic pain in all models. Associations for the other pathways were more selective and were concentrated in sleep duration and chronotype; metabolic-syndrome PRS was consistently associated with neuroticism. Cross-sample support was strongest for the association between obesity-mediated insulin-resistance PRS and morning chronotype.

**Conclusions:**

The overlap between T2D and psychiatric phenotypes was heterogeneous, with the strongest evidence for adiposity PRS and atypical depression and with more selective associations across other pathways. Considering heterogeneity in both T2D biology and psychiatric presentation may help clarify shared mechanisms.

## Plain Language Summary

Type 2 diabetes (T2D) and psychiatric disorders frequently co-occur. T2D is heterogeneous, with different mechanisms contributing to disease development in different people. Genetic variation that represents 8 mechanisms of T2D has been identified. In the UK Biobank study, we used genetic data to estimate the 8 mechanisms and explored how these mechanisms influence psychiatric disorders and related traits (including sleep problems and chronic pain). We found that specific T2D mechanisms were related to specific features of psychiatric disorders and related traits. The body fat mechanism was associated with atypical depression, anxiety, and chronic pain. The other mechanisms mainly influenced sleep traits.

Type 2 diabetes (T2D) and psychiatric disorders are 2 major public health challenges with an increasing global prevalence (1). T2D affects more than 10% of adults globally and is a leading cause of morbidity, disability, and premature mortality (2,3). Psychiatric conditions such as major depressive disorder (MDD) and generalized anxiety disorder (GAD) are among the top causes of years lived with disability worldwide (4). T2D and psychiatric conditions frequently co-occur and may interact through shared behavioral, metabolic, and biological mechanisms (1,5, 6, 7). Epidemiological studies estimate that up to 22% of individuals with psychiatric disorders also have T2D (1). Conversely, around one-third of individuals with T2D experience depressive symptoms (8), and this comorbidity contributes significantly to increased health care burden, reduced treatment adherence, and poor clinical outcomes (9, 10, 11).

Attention-deficit/hyperactivity disorder (ADHD), MDD, GAD, neuroticism, and insomnia have all been associated with an increased risk of subsequent T2D onset, even after accounting for lifestyle and cardiometabolic confounders (10,12,13). Additionally, T2D has also been associated with higher rates of chronic pain (7,14). Physical inactivity, stress, poor sleep, and an unhealthy diet are important contributors to both psychiatric morbidity and T2D (15, 16, 17) and may also arise as core symptoms or consequences of mental health conditions, further reinforcing this bidirectional relationship (18).

Recent studies suggest that shared genetics may play a role in comorbidity (10,19). A Mendelian randomization (MR) study supports a bidirectional causal relationship between MDD and T2D, with effects involving insulin resistance, adiposity, and body-fat distribution (7). This relationship may differ by depressive presentation: atypical depression, characterized here (20) by hypersomnia and weight gain, has been associated with a greater polygenic liability for T2D and insulin resistance than nonatypical depression (21). Cross-trait genome-wide association studies (GWASs) have also identified shared loci such as *IRS1*, *TCF7L2*, and *CDKAL1*, implicating insulin action, pancreatic function, and neural pathways (22,23). Genetically predicted insulin resistance has also been associated with the risk of schizophrenia (24).

The relationship between T2D and psychiatric disorders is further compounded by the marked heterogeneity of T2D. T2D arises through multiple pathophysiological mechanisms (25). Consequently, conventional genome-wide polygenic risk scores (PRSs), which aggregate genetic risk across pathogenic mechanisms, may obscure biologically distinct effects by combining heterogeneous genetic signals into a single measure (26). Suzuki *et al.* analyzed genetic associations with T2D in more than 2.5 million individuals and identified 1289 independent genetic signals mapping to 611 loci, which were organized into 8 mechanistic clusters (27). These clusters showed enrichment in genomic regulatory regions active in specific cell types, including pancreatic islets, adipocytes, and endothelial cells (27), and their PRSs differentially predicted vascular and renal outcomes (27). This framework provides a biologically informed lens with which to explore whether psychiatric traits might align more closely with specific T2D mechanisms.

Psychiatric phenotypes are also heterogeneous, and dimensional traits may capture biology extending across diagnostic boundaries (28). Neuroticism, mood instability, and anhedonia are dimensional traits that are heritable and genetically correlated with several psychiatric disorders (29, 30, 31), whereas risk-taking shares genetic liability with ADHD, bipolar disorder (BD), schizophrenia, and posttraumatic stress disorder (32,33). Sleep phenotypes provide complementary information because insomnia, sleep duration, and chronotype capture distinct aspects of sleep and circadian preference (34,35), which are also highly prevalent in psychiatric conditions. These phenotypes may reveal pathway-specific associations with T2D biology that would not be apparent from categorical diagnoses alone.

Here, we explored whether distinct components of T2D genetic liability show differential associations across psychiatric disorders and related dimensional, behavioral, and sleep phenotypes in the UK Biobank (UKB).

## Methods and Materials

### Summary Statistics

We used T2D-associated genetic variants identified by the multiancestry GWAS by Suzuki *et al.* (27). This previous study identified 1289 independent genome-wide significant variants and grouped them into 8 mechanistic clusters representing distinct biological pathways: beta cell dysfunction with positive proinsulin association (bcell+), beta cell dysfunction with negative proinsulin association (bcell–), insulin resistance mediated by obesity (obes), insulin resistance mediated by lipodystrophy (lipo), insulin resistance mediated by liver and lipid metabolism (liver), metabolic syndrome (metsyn), body fat (bfat), and residual glycemic variation (resgly). For this analyses, we used the index genetic variants and their assigned cluster labels and reported effect sizes exactly as reported in the original study, without further filtering or reclassification (Table S1).

### UKB Cohort

UKB is a large prospective study that recruited approximately 500,000 adults ages 40 to 69 years from across the United Kingdom between 2006 and 2010 (36). After recruitment, participants attended an assessment center where they completed detailed questionnaires covering sociodemographic characteristics, health history, and lifestyle behaviors and underwent a range of physical measurements. Follow-up information was obtained through repeat assessments, linkage to health records, and online questionnaires. Psychiatric data for this study came from 2 sources: the baseline assessment and the first Mental Health Questionnaire (MHQ). The first MHQ was an online survey, which was administered approximately 6 to 10 years after the baseline visit to a subset of participants to collect additional mental health information (37). All participants gave written informed consent, and ethical approval was provided by the National Health Service National Research Ethics Service (Ref: 21/NW/0157). This study was conducted under Project No. 71392 (principal investigator, RJS).

### Phenotypes

Phenotypes were derived from the UKB baseline touchscreen assessment (2006–2010) and the first MHQ (2016). Baseline phenotypes included anhedonia, assessed from the frequency of little interest or pleasure (#2060; ever vs. never), mood instability defined from an affirmative response to whether mood often goes up and down (#1920; binary), neuroticism (#20127; 0–12 Eysenck Personality Questionnaire-Revised Score; #871) (38), and risk-taking behavior (#2040; binary). Insomnia was assessed via the baseline touchscreen questionnaire (#1200), coded as usually/sometimes versus never/rarely, consistent with (39). Chronic pain (#6159) was assessed across 7 body sites, with chronicity defined as pain lasting ≥3 months. A continuous multisite chronic pain measure was derived as the number of sites meeting this criterion, excluding participants reporting “pain all over the body,” as previously described (40). This continuous measure is referred to as chronic pain throughout. A suicidality phenotype was categorized into 5 distinct severity levels as per Strawbridge *et al.* (41), and suicide deaths were ascertained using ICD-10 codes X60–X84; from these, a binary variable was derived capturing any suicidal ideation and/or behavior (severity 1–5 vs. 0). Sleep duration was derived from self-reported hours of sleep per 24 hours (#1160), with short sleep defined as <7 hours and long sleep as ≥9 hours, each compared with normal sleep of 7 to 8 hours. Chronotype was derived from (#1180), comparing definite morning and definite evening chronotypes separately with an intermediate group comprising participants reporting that they were more morning than evening or more evening than morning.

MDD was defined using 2 complementary approaches: 1) a lifetime history phenotype derived from the first MHQ items (MDD [MHQ]) (37) and 2) a baseline touchscreen phenotype following the validated UKB algorithm of (42) (MDD [Smith]). Atypical MDD comprised lifetime MDD with both hypersomnia and weight gain during the worst episode; remaining MDD cases were classified as nonatypical, with missing subtype-defining items excluded (20). Lifetime GAD and BD phenotypes were defined using lifetime history from the first MHQ responses (37).

Covariates included age at baseline assessment; type 1 diabetes (T1D) and T2D status based on self-report and/or medication use as per Eastwood *et al.* (43) (#293); broad ancestry category based on self-reported ethnicity as per Eastwood *et al.* (43); Townsend deprivation index (#22189); body mass index (BMI) calculated by the UKB (#21001); smoking status (#20116; current vs. former/never); and use of antidepressants, antipsychotics, mood-stabilizing, lipid-lowering, and antihypertensive medications (#20003). A cardiometabolic phenotype, ischemic heart disease (IHD), was coded based on self-reported heart attack and/or angina (#6150). Coding definitions and variable descriptions for all phenotypes are provided in Table S2.

### Genetic Data

UKB genetic data generation and quality control procedures have been described (44). Genotyping was performed using Affymetrix arrays and imputed to the Haplotype Reference Consortium and 1000 Genomes reference panels by the central UKB team. The analyses in this study were restricted to unrelated participants of self-reported White British ancestry in the discovery cohort and self-reported White European ancestry in the replication cohort. Related individuals were identified using kinship coefficients estimated by KING; one individual from each related pair was excluded at random (no pair was related closer than second degree). Variant-level quality control followed UKB recommendations, including the exclusion of poorly imputed variants and variants that were not present in the biobank, likely due to differences in imputation reference panels. Eleven variants were unavailable; their distribution across clusters is reported in Table S4.

### Polygenic Risk Scores

We generated cluster-specific PRSs for each of the 8 mechanistic T2D clusters (27). Scores were computed using PLINK (version 1.9) with the --score function, without clumping or linkage disequilibrium adjustment, consistent with the original partitioned scoring framework. Variants with minor allele frequency <1% or poor imputation quality (*r*^2^ < 0.5) or Hardy-Weinberg equilibrium (*p* < 10^-6^) were excluded. These PRSs were calculated for all participants in UKB with available genotype data and were standardized to a mean of zero and an SD of 1. The list of variants and their effect on T2D reported by Suzuki *et al.* (27) and used in each T2D cluster PRS is provided in Tables S5 to S12.

### Statistical Analysis

All statistical analyses were carried out in STATA 18 (StataCorp).

Pairwise Pearson’s correlations between the 8 cluster-specific PRSs were calculated to assess whether the scores represented independent aspects of T2D genetic risk.

Primary analyses were restricted to unrelated participants of self-reported White British ancestry, and individuals with T1D or T2D were excluded. We used linear regression for continuous traits and logistic regression for binary outcomes. Model 1 was adjusted for age at baseline, sex, genotyping chip, and the first 8 genetic principal components. Model 2 was additionally adjusted for Townsend deprivation index, BMI, and current smoking. Model 3 was additionally adjusted for antidepressant, antipsychotic, and mood-stabilizing medication use. Sensitivity analyses repeated model 1 after including participants with T1D/T2D. Because BMI may lie on the pathway between obesity-related genetic liability and psychiatric phenotypes, and psychotropic medication use may be both a consequence of psychiatric illness and a contributor to metabolic health, model 1 was considered the primary association model. Models 2 and 3 were interpreted as conditional sensitivity analyses rather than progressively less-confounded estimates. Secondary analyses were conducted in the mutually exclusive White European sample. The IHD positive control model was adjusted for age, sex, genotyping chip, the first 8 genetic principal components, Townsend deprivation index, BMI, current smoking, lipid-lowering and antihypertensive medication use, and T1D/T2D status. Benjamini-Hochberg false discovery rate (FDR) correction was applied jointly across all phenotype × PRSs × model combinations within each cohort; FDR-adjusted *p* values, hereafter referred to as *q* values, below .05 (*q* < .05) were considered statistically significant.

For phenotypes associated with two or more cluster-specific PRSs after FDR correction in at least model 1 or the diabetes-inclusive sensitivity analysis, the corresponding PRSs were entered simultaneously into secondary joint PRS analyses using the same covariate sequence. FDR correction was applied separately across all PRS coefficients and model specifications in these joint analyses.

## Results

Characteristics of the participants included in the discovery and replication analyses are presented in Table S3.

### Cluster-Specific PRS Capture Largely Nonoverlapping Linear Components of T2D Genetic Risk

Pairwise Pearson correlations between the 8 cluster-specific PRSs were uniformly small in both samples (maximum |*r*| ≈ 0.04) (Table S13), indicating limited overlap among the scores.

### Association of T2D Clusters With a Vascular Outcome and IHD

We examined IHD as a positive control and compared with European-ancestry estimates from Suzuki *et al.* (27) (Table S14).

Higher PRS-bfat and PRS-metsyn were associated with increased odds of IHD after FDR correction (PRS-bfat: odds ratio [OR] = 1.05, 95% CI [1.02–1.08], *q* = .004) (PRS-metsyn: OR = 1.04, 95% CI [1.01–1.07], *q* = .026). PRS-liver showed a positive association that was just above the FDR significance threshold (*q* = .051). No associations survived FDR correction in the smaller White European sample.

### The Associations of Adiposity-Related T2D Clusters With Psychiatric and Related Phenotypes

Associations of the PRS-bfat and PRS-obes scores with psychiatric and related phenotypes were examined in the White British cohort (Table 1), with corresponding White European analyses presented in Table S15.

**Table 1:** Associations of Adiposity-Related T2D Cluster-Specific Polygenic Risk Scores With Psychiatric and Related Phenotypes

PRS	Phenotype	Model 1			Model 1 + T1D/T2D			Model 2			Model 3		
		OR (95% CI) or β (SE)	*p*	*q*_FDR_	OR (95% CI) or β (SE)	*p*	*q*_FDR_	OR (95% CI) or β (SE)	*p*	*q*_FDR_	OR (95% CI) or β (SE)	*p*	*q*_FDR_
bfat	MDD MHQ	1.02 (1.01–1.04)	.001	.011^a^	1.02 (1.01–1.04)	.002	.016^a^	1.02 (1.01–1.04)	.007	.040^a^	1.02 (1.00–1.04)	.019	.085
	Atypical MDD	1.10 (1.05–1.16)	<.001	.001^a^	1.10 (1.05–1.15)	<.001	.002^a^	1.07 (1.02–1.13)	.009	.048^a^	1.08 (1.02–1.14)	.006	.040^a^
	Nonatypical MDD	1.02 (1.00–1.04)	.017	.080	1.02 (1.00–1.04)	.023	.096	1.02 (1.00–1.03)	.045	.146	1.02 (0.99–1.03)	.093	.239
	MDD Smith	1.02 (1.01–1.03)	.004	.027^a^	1.02 (1.00–1.03)	.008	.044^a^	1.02 (1.00–1.03)	.017	.079	1.02 (1.00–1.03)	.011	.057
	GAD	1.04 (1.02–1.06)	.001	.010^a^	1.04 (1.01–1.06)	.001	.012^a^	1.04 (1.01–1.06)	.002	.019^a^	1.04 (1.01–1.06)	.002	.020^a^
	Bipolar disorder	1.03 (0.99–1.08)	.183	.377	1.03 (0.99–1.08)	.165	.346	1.03 (0.98–1.07)	.280	.491	1.02 (0.97–1.07)	.485	.690
	Anhedonia	1.01 (1.00–1.02)	.004	.027^a^	1.01 (1.00–1.02)	.006	.039^a^	1.01 (0.99–1.02)	.060	.182	1.01 (0.99–1.02)	.088	.233
	Mood instability	1.01 (1.01–1.02)	<.001	.009^a^	1.01 (1.01–1.02)	<.001	.013^a^	1.01 (1.00–1.02)	.001	.009^a^	1.01 (1.00–1.02)	.001	.013^a^
	Risk-taking	0.99 (0.98–0.99)	.034	.124	0.99 (0.99–1.00)	.095	.244	0.99 (0.98–0.99)	.009	.048^a^	0.99 (0.98–0.99)	.003	.024^a^
	Insomnia	1.01 (1.00–1.02)	.003	.022^a^	1.01 (1.00–1.02)	.003	.023^a^	1.01 (1.00–1.02)	.011	.055	1.01 (1.00–1.02)	.017	.079
	Short sleep duration	1.01 (1.00–1.02)	.009	.049^a^	1.01 (1.00–1.02)	.009	.049^a^	1.01 (0.99–1.01)	.116	.274	1.01 (0.99–1.01)	.115	.274
	Long sleep duration	1.02 (1.01–1.04)	<.001	.002^a^	1.02 (1.01–1.03)	.001	.013^a^	1.02 (1.01–1.03)	.002	.016^a^	1.02 (1.01–1.03)	.003	.022^a^
	Morning chronotype	1.00 (0.99–1.01)	.859	.944	1.00 (0.99–1.01)	.662	.822	1.00 (0.99–1.01)	.986	.990	1.00 (0.99–1.01)	.823	.917
	Evening chronotype	0.99 (0.98–1.00)	.082	.222	0.99 (0.98–1.00)	.034	.124	0.99 (0.97–1.00)	.026	.102	0.99 (0.97–1.00)	.022	.093
	Suicidal behaviour	1.01 (0.99–1.02)	.286	.495	1.00 (0.99–1.02)	.421	.637	1.00 (0.99–1.02)	.424	.637	1.00 (0.99–1.02)	.515	.705
	Neuroticism	0.014 (0.006)	.012	.062	0.012 (0.006)	.036	.130	0.014 (0.006)	.013	.065	0.013 (0.006)	.019	.085
	Chronic pain	0.011 (0.002)	<.001	<.001^a^	0.010 (0.002)	<.001	<.001^a^	0.008 (0.002)	<.001	<.001^a^	0.007 (0.002)	<.001	<.001^a^
Obes	MDD MHQ	1.03 (1.01–1.04)	.001	.007^a^	1.03 (1.01–1.04)	<.001	.004^a^	0.99 (0.98–1.01)	.667	.826	1.00 (0.99–1.02)	.703	.847
	Atypical MDD	1.14 (1.09–1.20)	<.001	<.001^a^	1.15 (1.09–1.20)	<.001	<.001^a^	1.01 (0.96–1.06)	.741	.871	1.01 (0.95–1.07)	.788	.891
	Nonatypical MDD	1.02 (1.00–1.04)	.031	.117	1.02 (1.00–1.04)	.028	.110	0.99 (0.98–1.01)	.500	.697	0.99 (0.98–1.02)	.955	.980
	MDD Smith	1.02 (1.01–1.04)	<.001	.006^a^	1.02 (1.01–1.04)	<.001	.002^a^	1.00 (0.98–1.01)	.861	.944	1.00 (0.99–1.02)	.275	.491
	GAD	1.02 (1.00–1.05)	.039	.134	1.03 (1.00–1.05)	.018	.083	1.00 (0.98–1.03)	.761	.876	1.01 (0.99–1.04)	.398	.620
	Bipolar disorder	1.06 (1.01–1.11)	.021	.090	1.06 (1.01–1.11)	.019	.084	1.03 (0.98–1.08)	.235	.449	1.03 (0.98–1.08)	.272	.491
	Anhedonia	1.03 (1.02–1.03)	<.001	<.001^a^	1.03 (1.02–1.03)	<.001	<.001^a^	0.99 (0.99–1.00)	.243	.456	0.99 (0.99–1.01)	.576	.753
	Mood instability	1.02 (1.01–1.03)	<.001	<.001^a^	1.02 (1.01–1.03)	<.001	<.001^a^	0.99 (0.99–1.01)	.652	.818	1.00 (0.99–1.01)	.919	.977
	Risk-taking	1.02 (1.01–1.02)	<.001	.001^a^	1.01 (1.01–1.02)	<.001	.002^a^	1.00 (0.99–1.01)	.624	.792	1.00 (0.99–1.01)	.606	.780
	Insomnia	1.00 (0.99–1.01)	.750	.874	1.00 (0.99–1.01)	.663	.822	0.99 (0.98–0.99)	.003	.023^a^	0.99 (0.98–0.99)	.002	.018^a^
	Short sleep duration	1.02 (1.01–1.03)	<.001	<.001^a^	1.02 (1.01–1.03)	<.001	<.001^a^	0.99 (0.99–1.00)	.278	.491	0.99 (0.99–1.00)	.353	.573
	Long sleep duration	1.01 (0.99–1.02)	.322	.544	1.01 (0.99–1.02)	.118	.276	0.98 (0.97–0.99)	.001	.010^a^	0.98 (0.96–0.99)	.004	.026^a^
	Morning chronotype	1.03 (1.02–1.04)	<.001	<.001^a^	1.03 (1.02–1.04)	<.001	<.001^a^	1.02 (1.02–1.03)	<.001	<.001^a^	1.03 (1.02–1.03)	<.001	<.001^a^
	Evening chronotype	1.00 (0.99–1.01)	.953	.980	1.00 (0.99–1.01)	.683	.838	0.98 (0.96–0.99)	<.001	.001^a^	0.97 (0.96–0.99)	<.001	.001^a^
	Suicidal behaviour	1.01 (0.99–1.02)	.132	.296	1.01 (0.99–1.02)	.109	.266	0.99 (0.99–1.01)	.718	.858	1.00 (0.99–1.01)	.953	.980
	Neuroticism	−0.003 (0.006)	.622	.791	−0.004 (0.006)	.432	.643	−0.014 (0.006)	.014	.067	−0.008 (0.006)	.138	.306
	Chronic pain	0.022 (0.002)	<.001	<.001^a^	0.021 (0.002)	<.001	<.001^a^	−0.002 (0.002)	.330	.549	−0.002 (0.002)	.333	.550

Models: model 1 was adjusted for age, sex, genotyping chip, and the first 8 genetic principal components. Model 2 additionally adjusted for Townsend deprivation index, body mass index, and current smoking. Model 3 additionally adjusted for antidepressant, antipsychotic, and mood-stabilizing medication use. Participants with T1D or T2D were excluded from models 1–3; the sensitivity analysis repeated model 1 after including participants with T1D or T2D. Sample sizes varied by phenotype and model according to phenotype and covariate availability; phenotype-specific available *n* is reported in Table S3. Effect estimates are reported per 1-SD increase in PRS; *q* values < .05 were considered statistically significant. β is the linear regression coefficient.

bfat, body-fat distribution; FDR, false discovery rate; GAD, generalized anxiety disorder; MDD, major depressive disorder; MHQ, Mental Health Questionnaire; obes, obesity-mediated insulin resistance; OR, odds ratio; PRS, polygenic risk score; T1D, type 1 diabetes; T2D, type 2 diabetes.

a Indicates statistically significant results.

Associations with MDD were concentrated in the atypical subtype. In model 1, each standard deviation increase in PRS-bfat and PRS-obes was associated with 10% (OR = 1.10, 95% CI [1.05–1.16], *q* = .001) and 14% (OR = 1.14, 95% CI [1.09–1.20], *q* < .001) higher odds of atypical MDD, respectively. Neither PRS was associated with nonatypical MDD after FDR correction. PRS-bfat remained associated with GAD, mood instability, long sleep duration, and chronic pain across all 3 models, whereas associations with broader MDD definitions, anhedonia, insomnia, and short sleep were attenuated. PRS-bfat was also inversely associated with risk-taking in models 2 and 3.

PRS-obes showed a broader pattern of association in model 1, including MDD (MHQ), MDD (Smith), atypical MDD, anhedonia, mood instability, risk-taking, short sleep duration, and chronic pain. Most of these associations attenuated after adjustment in model 2. Morning chronotype remained associated across all models (model 3: OR = 1.03, 95% CI [1.02–1.03], *q* < .001), whereas inverse associations with insomnia, long sleep duration, and evening chronotype were observed in models 2 and 3.

The sensitivity analyses (including participants with diabetes) were consistent with model 1. In the White European sample, consistency was strongest for PRS-obes and morning chronotype, which remained associated across all models (model 3: OR = 1.05, 95% CI [1.02–1.07], *q* = .007). No PRS-bfat association survived FDR correction, although several estimates were directionally consistent.

In the joint analyses, PRS-bfat remained associated with atypical MDD across all models after modeling PRS-bfat and PRS-obes together, whereas the association with PRS-obes was confined to model 1 and the diabetes-inclusive sensitivity analysis (Table S16). A similar attenuation of PRS-obes was observed for the broader MDD phenotypes.

### Associations of Glycemic Clusters With Psychiatric and Related Phenotypes

The 2 beta-cell scores and PRS-resgly were examined in the White British sample (Table 2), with corresponding White European analyses presented in Table S17.

**Table 2:** Associations of Glycemic T2D Cluster-Specific Polygenic Risk Scores With Psychiatric and Related Phenotypes

PRS	Phenotype	Model 1			Model 1 + T1D/T2D			Model 2			Model 3		
		OR (95% CI) or β (SE)	*p*	*q*_FDR_	OR (95% CI) or β (SE)	*p*	*q*_FDR_	OR (95% CI) or β (SE)	*p*	*q*_FDR_	OR (95% CI) or β (SE)	*p*	*q*_FDR_
bcell+	MDD MHQ	0.98 (0.97–0.99)	.039	.134	0.98 (0.97–0.99)	.038	.134	0.99 (0.98–1.01)	.277	.491	0.99 (0.98–1.01)	.403	.623
	Atypical MDD	0.97 (0.92–1.01)	.157	.337	0.96 (0.92–1.01)	.131	.295	1.00 (0.95–1.06)	.879	.948	1.02 (0.96–1.07)	.604	.780
	Nonatypical MDD	0.98 (0.97–1.00)	.056	.176	0.98 (0.97–0.99)	.039	.134	0.99 (0.97–1.01)	.190	.386	0.99 (0.98–1.01)	.411	.633
	MDD Smith	0.99 (0.98–1.00)	.153	.330	0.99 (0.98–1.00)	.065	.190	0.99 (0.98–1.01)	.425	.637	0.99 (0.98–1.01)	.465	.674
	GAD	0.99 (0.97–1.01)	.353	.573	0.99 (0.97–1.01)	.261	.479	0.99 (0.97–1.01)	.475	.682	0.99 (0.97–1.02)	.495	.695
	Bipolar disorder	0.99 (0.95–1.04)	.738	.870	0.99 (0.94–1.03)	.629	.796	0.99 (0.95–1.04)	.830	.922	0.99 (0.94–1.04)	.634	.801
	Anhedonia	0.99 (0.98–0.99)	.007	.041^a^	0.99 (0.98–0.99)	.002	.019^a^	0.99 (0.99–1.00)	.208	.413	0.99 (0.99–1.00)	.304	.518
	Mood instability	0.99 (0.98–0.99)	.003	.022^a^	0.99 (0.98–0.99)	<.001	.005^a^	0.99 (0.99–1.00)	.088	.233	0.99 (0.99–1.00)	.107	.264
	Risk-taking	0.99 (0.99–1.00)	.296	.506	0.99 (0.99–1.00)	.219	.430	0.99 (0.99–1.01)	.689	.840	0.99 (0.99–1.01)	.530	.715
	Insomnia	0.99 (0.99–1.01)	.545	.725	0.99 (0.99–1.00)	.469	.676	1.00 (0.99–1.01)	.877	.947	1.00 (0.99–1.01)	.865	.944
	Short sleep duration	0.99 (0.99–1.00)	.236	.449	0.99 (0.99–1.00)	.119	.276	1.00 (0.99–1.01)	.966	.980	1.00 (0.99–1.01)	.821	.917
	Long sleep duration	0.98 (0.96–0.99)	<.001	.001^a^	0.98 (0.97–0.99)	<.001	<.001^a^	0.98 (0.97–0.99)	.002	.019	0.98 (0.97–0.99)	.002	.019
	Morning chronotype	1.01 (1.00–1.01)	.174	.361	1.00 (1.00–1.01)	.413	.633	1.01 (1.00–1.02)	.056	.176	1.01 (1.00–1.02)	.044	.146
	Evening chronotype	0.97 (0.96–0.99)	<.001	<.001^a^	0.97 (0.96–0.99)	<.001	<.001^a^	0.98 (0.97–0.99)	<.001	.004	0.98 (0.97–0.99)	.001	.010
	Suicidal behaviour	0.99 (0.98–1.01)	.370	.587	0.99 (0.98–1.01)	.277	.491	0.99 (0.98–1.01)	.605	.780	0.99 (0.99–1.01)	.858	.944
	Neuroticism	−0.009 (0.006)	.11	.266	−0.011 (0.006)	.057	.177	−0.007 (0.006)	.240	.452	−0.007 (0.006)	.240	.452
	Chronic pain	−0.007 (0.002)	<.001	<.001^a^	−0.009 (0.002)	<.001	<.001^a^	−0.003 (0.002)	.098	.248	−0.003 (0.002)	.130	.294
bcell−	MDD MHQ	1.00 (0.99–1.02)	.980	.987	0.99 (0.98–1.01)	.708	.851	1.00 (0.99–1.02)	.694	.842	1.00 (0.99–1.02)	.564	.741
	Atypical MDD	0.98 (0.93–1.02)	.327	.545	0.98 (0.93–1.03)	.382	.602	0.98 (0.93–1.03)	.488	.691	0.98 (0.93–1.03)	.441	.653
	Nonatypical MDD	1.00 (0.99–1.02)	.744	.872	0.99 (0.98–1.02)	.956	.980	1.00 (0.99–1.02)	.561	.741	1.01 (0.99–1.02)	.477	.682
	MDD Smith	0.99 (0.98–1.01)	.240	.452	0.99 (0.98–1.00)	.111	.268	0.99 (0.98–1.01)	.412	.633	0.99 (0.98–1.01)	.465	.674
	GAD	1.01 (0.98–1.03)	.612	.783	1.00 (0.98–1.03)	.674	.830	1.01 (0.99–1.03)	.481	.686	1.01 (0.99–1.04)	.281	.491
	Bipolar disorder	1.05 (0.99–1.10)	.056	.176	1.04 (0.99–1.09)	.101	0.252	1.05 (1.00–1.10)	.041	.139	1.06 (1.01–1.11)	.024	.098
	Anhedonia	0.99 (0.99–1.01)	.672	.830	0.99 (0.99–1.00)	.365	.587	1.00 (0.99–1.01)	.602	.780	1.00 (0.99–1.01)	.295	.506
	Mood instability	0.99 (0.98–0.99)	.005	.031^a^	0.99 (0.98–0.99)	.001	.009^a^	0.99 (0.99–0.99)	.045	.146	0.99 (0.99–1.00)	.062	.186
	Risk-taking	0.99 (0.99–1.00)	.251	.467	0.99 (0.99–1.00)	.142	.311	0.99 (0.99–1.01)	.514	.705	0.99 (0.99–1.00)	.417	.634
	Insomnia	0.99 (0.99–1.00)	.189	.385	0.99 (0.99–1.00)	.264	.483	0.99 (0.99–1.00)	.403	.623	0.99 (0.99–1.00)	.389	.608
	Short sleep duration	0.99 (0.99–1.00)	.167	.348	0.99 (0.99–1.00)	.091	.237	0.99 (0.99–1.01)	.531	.715	0.99 (0.99–1.01)	.617	.787
	Long sleep duration	0.99 (0.98–1.00)	.253	.469	0.99 (0.98–1.00)	.060	.182	0.99 (0.98–1.01)	.589	.769	0.99 (0.99–1.01)	.765	.876
	Morning chronotype	1.00 (1.00–1.01)	.283	.491	1.00 (1.00–1.01)	.424	.637	1.01 (1.00–1.01)	.184	.378	1.01 (1.00–1.01)	.189	.385
	Evening chronotype	0.99 (0.98–1.00)	.044	.146	0.99 (0.98–1.00)	.080	.220	0.99 (0.98–1.00)	.129	.294	0.99 (0.98–1.00)	.160	.338
	Suicidal behaviour	0.99 (0.99–1.01)	.613	.783	0.99 (0.98–1.01)	.486	.690	0.99 (0.99–1.01)	.755	.876	0.99 (0.99–1.01)	.874	.947
	Neuroticism	−0.008 (0.006)	.15	.325	−0.01 (0.006)	.083	.222	−0.007 (0.006)	.249	.464	−0.005 (0.006)	.389	.608
	Chronic pain	−0.007 (0.002)	<.001	<.001^a^	−0.008 (0.002)	<.001	<.001^a^	−0.004 (0.002)	.013	.064	−0.004 (0.002)	.014	.067
Resgly	MDD MHQ	0.99 (0.98–1.01)	.964	.980	0.99 (0.98–1.01)	.689	.840	0.99 (0.98–1.01)	.765	.876	0.99 (0.98–1.01)	.854	.944
	Atypical MDD	1.06 (1.01–1.11)	.024	.097	1.05 (1.00–1.10)	.034	.124	1.04 (0.99–1.10)	.104	.259	1.03 (0.97–1.09)	.352	.573
	Nonatypical MDD	0.99 (0.98–1.01)	.533	.716	0.99 (0.98–1.01)	.429	.642	0.99 (0.98–1.01)	.509	.705	0.99 (0.98–1.01)	.694	.842
	MDD Smith	1.01 (0.99–1.02)	.130	.294	1.01 (0.99–1.02)	.135	.302	1.01 (0.99–1.02)	.166	.347	1.01 (0.99–1.03)	.093	.239
	GAD	1.01 (0.99–1.04)	.236	.449	1.01 (0.99–1.03)	.276	.491	1.01 (0.99–1.04)	.259	.477	1.01 (0.99–1.04)	.335	.552
	Bipolar disorder	1.00 (0.96–1.05)	.887	.954	0.99 (0.95–1.04)	.863	.944	1.00 (0.96–1.05)	.864	.944	0.99 (0.94–1.04)	.724	.858
	Anhedonia	1.01 (0.99–1.01)	.160	.338	1.00 (0.99–1.01)	.295	.506	1.00 (0.99–1.01)	.323	.544	1.01 (0.99–1.02)	.146	.319
	Mood instability	1.00 (0.99–1.01)	.423	.637	1.00 (0.99–1.01)	.490	.692	1.00 (0.99–1.01)	.689	.840	1.00 (0.99–1.01)	.516	.705
	Risk-taking	0.99 (0.98–0.99)	.028	.109	0.99 (0.98–0.99)	.018	.080	0.99 (0.98–0.99)	.017	.080	0.99 (0.98–0.99)	.022	.093
	Insomnia	1.00 (0.99–1.01)	.221	.431	1.00 (0.99–1.01)	.280	.491	1.00 (0.99–1.01)	.293	.505	1.00 (0.99–1.01)	.278	.491
	Short sleep duration	0.99 (0.99–1.01)	.751	.874	0.99 (0.99–1.00)	.437	.648	0.99 (0.99–1.01)	.526	.713	0.99 (0.99–1.01)	.783	.891
	Long sleep duration	1.00 (0.99–1.01)	.806	.906	0.99 (0.99–1.01)	.955	.980	0.99 (0.99–1.01)	.928	.977	0.99 (0.99–1.01)	.964	.980
	Morning chronotype	1.01 (1.00–1.02)	.002	.019^a^	1.01 (1.00–1.02)	.007	.040^a^	1.01 (1.00–1.02)	.003	.022^a^	1.01 (1.00–1.02)	.007	.040^a^
	Evening chronotype	1.00 (0.98–1.01)	.387	.608	0.99 (0.98–1.01)	.282	.491	0.99 (0.98–1.01)	.322	.544	0.99 (0.98–1.00)	.196	.395
	Suicidal behaviour	1.00 (0.99–1.01)	.722	.858	1.00 (0.99–1.01)	.862	.944	1.00 (0.99–1.01)	.867	.944	1.00 (0.99–1.01)	.785	.891
	Neuroticism	0.007 (0.006)	.206	.412	0.005 (0.006)	.340	.559	0.006 (0.006)	.254	.469	0.008 (0.006)	.141	.310
	Chronic pain	0.001 (0.002)	.711	.853	−0.001 (0.002)	.927	.977	−0.001 (0.002)	.700	.846	−0.001 (0.002)	.877	.947

Models: model 1 was adjusted for age, sex, genotyping chip, and the first 8 genetic principal components. Model 2 additionally adjusted for Townsend deprivation index, body mass index, and current smoking. Model 3 additionally adjusted for antidepressant, antipsychotic, and mood-stabilizing medication use. Participants with T1D or T2D were excluded from models 1–3; the sensitivity analysis repeated model 1 after including participants with T1D or T2D. Sample sizes varied by phenotype and model according to phenotype and covariate availability; phenotype-specific available *n* is reported in Table S3. Effect estimates are reported per 1-SD increase in PRS; *q* values < .05 were considered statistically significant. β is the linear regression coefficient.

bcell+, beta-cell dysfunction with positive proinsulin association; bcell−, beta-cell dysfunction with negative proinsulin association; FDR, false discovery rate; GAD, generalized anxiety disorder; MDD, major depressive disorder; MHQ, Mental Health Questionnaire; OR, odds ratio; PRS, polygenic risk score; resgly, residual glycemic variation; T1D, type 1 diabetes; T2D, type 2 diabetes.

a Indicates statistically significant results.

PRS-bcell+ was consistently associated with sleep phenotypes. Higher PRS-bcell+ was associated with lower odds of long sleep duration and evening chronotype across all models (model 1: OR = 0.98, 95% CI [0.96–0.99], *q* = .001; OR = 0.97, 95% CI [0.96–0.99], *q* < .001, respectively), with both associations persisting in model 3. Inverse associations with anhedonia, mood instability, and chronic pain were confined to model 1.

PRS-bcell– was inversely associated with mood instability (OR = 0.99, 95% CI [0.98–0.99], *q* = .031) and chronic pain (β = −0.007, SE = 0.002, *q* < .001) in model 1, but neither remained significant in models 2 or 3. Neither beta-cell score was associated with categorical psychiatric diagnoses after FDR correction.

PRS-resgly was associated only with morning chronotype, with the association persisting despite model 3 adjustment (OR = 1.01, 95% CI [1.00–1.02], *q* = .040).

The sensitivity analysis closely mirrored model 1. In the White European sample, most estimates did not survive FDR correction; only the inverse PRS-bcell+ association with long sleep duration was significant.

In the joint models, the opposing associations of PRS-bfat and PRS-bcell+ with long sleep duration persisted across all models. For mood instability, beta-cell associations attenuated after additional covariate adjustment, whereas PRS-bfat remained associated (Table S16).

### Associations of Metabolic and Lipid T2D Clusters With Psychiatric and Related Traits

Among the remaining pathways, PRS-metsyn, PRS-lipo, and PRS-liver were examined in White British participants (Table 3), with analyses in the White European sample (Table S18).

**Table 3:** Associations of Metabolic and Lipid-Related T2D Cluster-Specific Polygenic Risk Scores With Psychiatric and Related Phenotypes

PRS	Phenotype	Model 1			Model 1 + T1D/T2D			Model 2			Model 3		
		OR (95% CI) or β (SE)	*p*	*q*_FDR_	OR (95% CI) or β (SE)	*p*	*q*_FDR_	OR (95% CI) or β (SE)	*p*	*q*_FDR_	OR (95% CI) or β (SE)	*p*	*q*_FDR_
Metsyn	MDD MHQ	1.01 (0.99–1.02)	.205	.411	1.01 (0.99–1.02)	.222	.431	1.01 (0.99–1.03)	.071	.201	1.01 (0.99–1.03)	.079	.219
	Atypical MDD	1.02 (0.97–1.07)	.356	.576	1.02 (0.97–1.07)	.430	.642	1.04 (0.99–1.10)	.119	.276	1.02 (0.96–1.08)	.515	.705
	Nonatypical MDD	1.01 (0.99–1.02)	.451	.664	1.01 (0.99–1.02)	.452	.664	1.01 (0.99–1.03)	.214	.423	1.01 (0.99–1.03)	.110	.266
	MDD Smith	1.00 (0.99–1.02)	.639	.803	1.01 (0.99–1.02)	.457	.667	1.01 (0.99–1.02)	.370	.587	1.01 (0.99–1.02)	.446	.658
	GAD	1.02 (0.99–1.04)	.096	.244	1.02 (0.99–1.04)	.115	.274	1.02 (0.99–1.04)	.083	.222	1.02 (0.99–1.05)	.081	.222
	Bipolar disorder	1.01 (0.96–1.05)	.777	.888	1.00 (0.96–1.05)	.971	.980	1.01 (0.96–1.06)	.718	.858	0.99 (0.95–1.05)	.995	.997
	Anhedonia	1.01 (0.99–1.01)	.178	.368	1.01 (0.99–1.01)	.208	.413	1.01 (1.00–1.02)	.014	.070	1.01 (1.00–1.02)	.004	.026
	Mood instability	1.01 (1.00–1.01)	.034	.124	1.01 (1.00–1.01)	.025	.100	1.01 (1.00–1.02)	.002	.019^a^	1.01 (1.01–1.02)	.001	.007
	Risk-taking	1.00 (0.99–1.01)	.477	.682	1.00 (0.99–1.01)	.399	.620	1.01 (0.99–1.01)	.164	.346	1.01 (0.99–1.01)	.195	.394
	Insomnia	1.01 (1.00–1.02)	.015	.071	1.01 (1.00–1.02)	.017	.080	1.01 (1.00–1.02)	.006	.039^a^	1.01 (1.00–1.02)	.005	.031
	Short sleep duration	1.01 (0.99–1.01)	.065	.190	1.01 (0.99–1.01)	.106	.262	1.01 (1.00–1.02)	.009	.049^a^	1.01 (1.00–1.02)	.010	.051
	Long sleep duration	1.01 (1.00–1.02)	.046	.148	1.01 (0.99–1.02)	.052	.164	1.02 (1.00–1.03)	.008	.046^a^	1.02 (1.00–1.03)	.009	.049^a^
	Morning chronotype	1.01 (1.00–1.02)	.051	.163	1.01 (1.00–1.02)	.033	.124	1.01 (1.00–1.02)	.037	.132	1.01 (1.00–1.02)	.075	.209
	Evening chronotype	1.00 (0.99–1.01)	.969	.980	1.00 (0.99–1.01)	.724	.858	1.00 (0.99–1.02)	.549	.728	1.00 (0.99–1.02)	.564	.741
	Suicidal behaviour	1.01 (0.99–1.02)	.147	.319	1.01 (0.99–1.02)	.129	.294	1.01 (0.99–1.02)	.090	.236	1.01 (0.99–1.02)	.068	.195
	Neuroticism	0.020 (0.006)	<.001	.004^a^	0.021 (0.006)	<.001	.002^a^	0.021 (0.006)	<.001	.003^a^	0.024 (0.006)	<.001	<.001^a^
	Chronic pain	−0.001 (0.002)	.659	.822	−0.001 (0.002)	.414	.633	0.002 (0.002)	.160	.338	0.003 (0.002)	.110	.266
Lipo	MDD MHQ	0.99 (0.98–1.01)	.931	.977	0.99 (0.98–1.01)	.742	.871	1.00 (0.99–1.02)	.604	.780	1.00 (0.99–1.02)	.654	.819
	Atypical MDD	0.98 (0.94–1.03)	.467	.675	0.97 (0.93–1.02)	.248	.464	1.02 (0.97–1.07)	.515	.705	1.01 (0.95–1.06)	.813	.912
	Nonatypical MDD	1.01 (0.99–1.02)	.530	.715	1.00 (0.99–1.02)	.610	.783	1.01 (0.99–1.02)	.369	.587	1.01 (0.99–1.03)	.321	.544
	MDD Smith	1.03 (0.99–1.07)	.116	.274	1.02 (0.99–1.06)	.228	.442	1.03 (0.99–1.07)	.112	.269	1.00 (0.99–1.02)	.554	.733
	GAD	1.01 (0.99–1.03)	.368	.587	1.01 (0.99–1.03)	.536	.718	1.01 (0.99–1.04)	.217	.428	1.01 (0.99–1.03)	.460	.67
	Bipolar disorder	1.01 (0.96–1.05)	.819	.917	1.01 (0.96–1.05)	.824	.917	1.02 (0.97–1.06)	.513	.705	1.01 (0.96–1.06)	.722	.858
	Anhedonia	0.99 (0.99–1.01)	.926	.977	1.00 (0.99–1.01)	.963	.980	1.00 (0.99–1.01)	.268	.487	1.00 (0.99–1.01)	.752	.874
	Mood instability	1.00 (0.99–1.01)	.933	.977	1.00 (0.99–1.01)	.787	.891	1.00 (0.99–1.01)	.267	.486	1.00 (0.99–1.01)	.565	.741
	Risk-taking	1.00 (0.99–1.01)	.342	.560	1.00 (0.99–1.01)	.417	.634	1.01 (0.99–1.01)	.140	.309	1.01 (0.99–1.01)	.077	.214
	Insomnia	0.99 (0.99–1.00)	.494	.695	0.99 (0.99–1.01)	.540	.722	0.99 (0.99–1.01)	.762	.876	0.99 (0.99–1.01)	.543	.724
	Short sleep duration	1.01 (0.99–1.01)	.059	.181	1.01 (1.00–1.02)	.041	.139	1.01 (1.00–1.02)	.002	.017^a^	1.01 (1.00–1.02)	.005	.03^a^
	Long sleep duration	1.01 (0.99–1.02)	.326	.545	1.00 (0.99–1.02)	.497	.696	1.01 (0.99–1.02)	.072	.204	1.00 (0.99–1.02)	.220	.431
	Morning chronotype	1.01 (1.00–1.02)	.006	.040^a^	1.01 (1.00–1.02)	.004	.026^a^	1.01 (1.00–1.02)	.003	.024^a^	1.01 (1.01–1.02)	.001	.01^a^
	Evening chronotype	0.99 (0.98–1.01)	.325	.545	0.99 (0.98–1.01)	.332	.550	1.00 (0.99–1.01)	.853	.944	1.00 (0.99–1.01)	.948	.98
	Suicidal behaviour	0.99 (0.99–1.01)	.636	.802	0.99 (0.98–1.01)	.360	.581	0.99 (0.99–1.01)	.936	.979	0.99 (0.99–1.01)	.759	.876
	Neuroticism	−0.001 (0.006)	.806	.906	−0.001 (0.006)	.919	.977	0.001 (0.006)	.964	.980	−0.003 (0.006)	.604	.78
	Chronic pain	−0.005 (0.002)	.007	.04^a^	−0.005 (0.002)	.008	.045^a^	−0.001 (0.002)	.751	.874	−0.001 (0.002)	.522	.71
Liver	MDD MHQ	0.99 (0.98–1.01)	.960	.980	1.00 (0.99–1.02)	.912	.976	0.99 (0.98–1.01)	.787	.891	0.99 (0.98–1.01)	.734	.868
	Atypical MDD	1.06 (1.01–1.11)	.022	.092	1.05 (1.00–1.10)	.034	.124	1.06 (1.01–1.12)	.021	.090	1.06 (1.01–1.13)	.031	.117
	Nonatypical MDD	1.00 (0.98–1.02)	.929	.977	1.00 (0.99–1.02)	.698	.845	0.99 (0.98–1.02)	.918	.977	0.99 (0.98–1.02)	.801	.904
	MDD Smith	1.00 (0.99–1.01)	.966	.980	0.99 (0.99–1.01)	.909	.974	0.99 (0.99–1.01)	.943	.980	1.01 (0.99–1.02)	.455	.666
	GAD	1.00 (0.98–1.03)	.676	.831	1.01 (0.99–1.03)	.500	.697	1.00 (0.98–1.03)	.764	.876	1.01 (0.98–1.03)	.522	.71
	Bipolar disorder	0.97 (0.92–1.01)	.130	.294	0.96 (0.92–1.01)	.089	.235	0.96 (0.92–1.01)	.098	.247	0.97 (0.93–1.02)	.233	.448
	Anhedonia	0.99 (0.99–1.00)	.159	.338	0.99 (0.99–1.00)	.075	.209	0.99 (0.99–1.00)	.078	.217	0.99 (0.99–1.00)	.230	.444
	Mood instability	0.99 (0.99–0.99)	.043	.144	0.99 (0.99–0.99)	.025	.100	0.99 (0.99–0.99)	.012	.059	0.99 (0.99–0.99)	.036	.13
	Risk-taking	0.99 (0.99–1.00)	.119	.276	0.99 (0.99–1.00)	.069	.197	0.99 (0.99–1.00)	.067	.194	0.99 (0.99–1.00)	.092	.237
	Insomnia	1.01 (1.00–1.02)	.048	.154	1.01 (0.99–1.01)	.061	.183	1.01 (0.99–1.01)	.067	.194	1.01 (1.00–1.02)	.042	.143
	Short sleep duration	0.99 (0.99–1.00)	.067	.194	0.99 (0.99–1.00)	.099	.248	0.99 (0.98–0.99)	.034	.124	0.99 (0.98–0.99)	.030	.117
	Long sleep duration	1.01 (0.99–1.02)	.059	.180	1.01 (0.99–1.02)	.058	.179	1.01 (0.99–1.02)	.088	.233	1.01 (1.00–1.03)	.042	.143
	Morning chronotype	0.99 (0.98–1.00)	.021	.090	0.99 (0.98–1.00)	.022	.093	0.99 (0.98–1.00)	.024	.097	0.99 (0.98–1.00)	.023	.095
	Evening chronotype	1.01 (1.00–1.03)	.044	.146	1.01 (1.00–1.02)	.038	.133	1.01 (1.00–1.02)	.063	.186	1.01 (1.00–1.02)	.082	.222
	Suicidal behaviour	1.00 (0.99–1.01)	.957	.980	1.00 (0.99–1.01)	.872	.947	0.99 (0.99–1.01)	.969	.980	0.99 (0.99–1.01)	.951	.98
	Neuroticism	0.001 (0.006)	.928	.977	−0.001 (0.006)	.999	.999	−0.001 (0.006)	.898	.964	0.003 (0.006)	.660	.822
	Chronic pain	0.002 (0.002)	.279	.491	0.002 (0.002)	.369	.587	0.001 (0.002)	.515	.705	0.002 (0.002)	.372	.588

Models: model 1 was adjusted for age, sex, genotyping chip, and the first 8 genetic principal components. Model 2 additionally adjusted for Townsend deprivation index, body mass index, and current smoking. Model 3 additionally adjusted for antidepressant, antipsychotic, and mood-stabilizing medication use. Participants with T1D or T2D were excluded from models 1–3; the sensitivity analysis repeated model 1 after including participants with T1D or T2D. Sample sizes varied by phenotype and model according to phenotype and covariate availability; phenotype-specific available *n* is reported in Table S3. Effect estimates are reported per 1-SD increase in PRS; *q* values < .05 were considered statistically significant. β is the linear regression coefficient.

FDR, false discovery rate; GAD, generalized anxiety disorder; lipo, lipodystrophy-mediated insulin resistance; liver, liver and lipid metabolism; MDD, major depressive disorder; metsyn, metabolic syndrome; MHQ, Mental Health Questionnaire; OR, odds ratio; PRS, polygenic risk score; T1D, type 1 diabetes; T2D, type 2 diabetes.

a Indicates statistically significant results.

PRS-metsyn was consistently associated with neuroticism, which persisted across all models (model 1: β = 0.020, SE = 0.006, *q* = .004; model 3: β = 0.024, SE = 0.006, *q* < .001). Mood instability, insomnia, and long sleep duration were also associated in models 2 and 3, with short sleep in model 2 and anhedonia in model 3.

PRS-lipo was associated with morning chronotype across all models (model 1: OR = 1.01, 95% CI [1.00–1.02], *q* = .040; model 3: OR = 1.01, 95% CI [1.01–1.02], *q* = .010) and with short sleep duration in models 2 and 3; its inverse association with chronic pain was confined to model 1. No PRS-liver association survived FDR correction.

No association survived FDR correction in the White European sample, although the directions of PRS-metsyn with neuroticism and PRS-lipo with morning chronotype and short sleep were consistent with the findings in White British individuals.

### Overview of T2D Clusters Associations Across Psychiatric Phenotypes

Figure 1 provides a visual overview of the associations between the 8 mechanistic T2D clusters and psychiatric and related phenotypes (z scores and FDR-corrected *q*-values are reported in Table S19).

**Figure 1:**
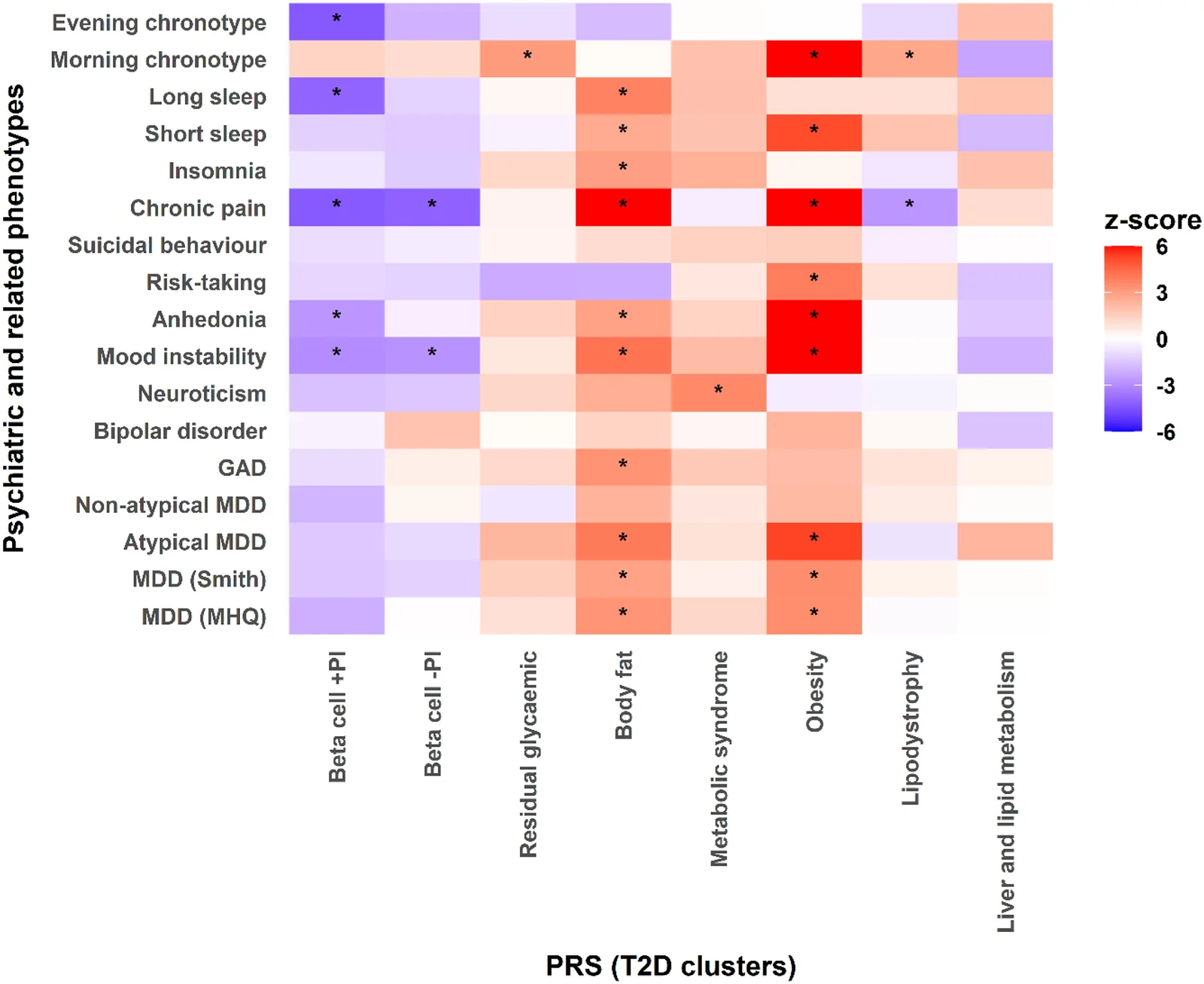
Heat map of model 1 associations between 8 mechanistic T2D cluster-specific PRSs and 17 psychiatric and related phenotypes in the White British sample. Cells show *z* scores, with red color indicating positive and blue indicating negative associations. ∗ denotes associations surviving FDR correction (*q* < .05). Model 1 was adjusted for age, sex, genotyping chip, and the first 8 genetic principal components; participants with diabetes were excluded. *z* scores correspond to Wald statistics for binary outcomes and *t* statistics for continuous outcomes; given the large sample sizes, the latter approximate standard normal statistics. For visualization, the color scale was capped at *z* = ±6; exact *z* scores and FDR-adjusted *q* values are provided in Table S19. FDR, false discovery rate; GAD, generalized anxiety disorder; MDD, major depressive disorder; MHQ, Mental Health Questionnaire; PI, proinsulin; PRS, polygenic risk score; T2D, type 2 diabetes.

## Discussion

We examined whether specific mechanisms of T2D (defined by cluster-specific PRS) were associated with specific psychiatric and related phenotypes. Three main patterns stood out. Firstly, adiposity-related mechanisms showed the broadest range of associations, particularly with atypical MDD. Secondly, beta-cell driven glycemic pathways were inversely associated with selected affective, pain, and sleep-related phenotypes. Third, the metabolic- and lipid-related pathways showed specific associations with neuroticism, sleep duration, and chronotype, whereas the liver/lipid pathway showed little evidence of association. Taken together, these findings indicate that associations between T2D and psychiatric phenotypes are mechanism-specific, consistent with recent work emphasizing the biological heterogeneity of T2D (27,45).

### Adiposity-Driven Metabolic Risk as a Key Interface With Mental Health

Adiposity-related genetic risks (PRS-bfat and PRS-obes) were associated with atypical MDD, but neither was associated with nonatypical MDD. Further analyses highlighted that the atypical-depression signal was more strongly aligned with body-fat distribution than with obesity per se. This is consistent with a greater T2D and insulin-resistance polygenic liability in atypical depression (21), and MR evidence implicating adiposity and insulin resistance in the T2D-depression relationship (7).

PRS-bfat was associated with a wide range of traits, despite full adjustment, specifically GAD, mood instability, long sleep duration, and chronic pain. PRS-obes associations were attenuated with full adjustment. These models are complementary because BMI may partly mediate obesity-related genetic liability rather than acting solely as a confounder. Morning chronotype was the notable exception, persisting for PRS-obes across all models and in White Europeans. Metabolic and circadian regulation are closely linked through adipose function, glucose metabolism, and insulin sensitivity (46). The adjusted inverse associations with evening chronotype, insomnia, and long sleep duration should therefore be viewed as conditional associations rather than evidence of a protective sleep profile.

The inverse PRS-bfat association with risk-taking emerged only after adjustment and contrasted with the positive effects of PRS-BMI and risk-taking (33). Its emergence only after additional adjustment raises the possibility of over-adjustment or collider bias, whereas UKB healthy-volunteer selection may also contribute to bias (47,48). Therefore, the inverse association should be considered exploratory rather than evidence that adiposity-related genetic liability directly reduces risk-taking.

MDD, anhedonia, and mood instability partly overlap and should not be treated as independent confirmation, but their pattern is compatible with adiposity mechanisms influencing a range of affective dimensions, in keeping with prior evidence linking T2D with MDD and anxiety through insulin and neural pathways (22). Adipose tissue is also an endocrine and immunologic organ, and variation in fat storage and distribution can influence inflammation, adipokines, neuroendocrine signaling, and central insulin sensitivity (49,50), processes relevant to the weight, energy, and sleep disturbances of atypical depression.

The persistent association with chronic pain adds a somatic dimension, consistent with metabolic, inflammatory, and mechanical links between adiposity and pain (51).

### Beta-Cell Centered Glycemic Pathways Play a Limited Role

In contrast, beta-cell and residual glycemic scores were associated only with sleep duration and chronotype. Pancreatic islets possess intrinsic molecular clocks that coordinate rhythmic insulin secretion, and clock disruption impairs beta-cell function and glucose regulation (52,53). These results are compatible with shared genetic influences on metabolic and circadian regulation, and not evidence that greater glycemic liability produces a healthier sleep profile.

Inverse beta-cell associations with anhedonia, mood instability, and chronic pain were small and did not survive further adjustments. Peripheral insulin resistance has been linked to reduced cerebral glucose metabolism and poorer cognition (54,55), whereas experimental work supports metabolic influences on pain processing (56), but neither directly explains the inverse beta-cell estimates. Biases from excluding participants with diabetes, pleiotropy, residual confounding, or chance remain plausible.

### Lipid Regulation Influences Specific Features of Mental Illness

Among the remaining pathways, PRS-metsyn was consistently associated with neuroticism, consistent with observational evidence linking higher neuroticism to metabolic syndrome (57). Because this score integrates insulin resistance, adiposity, dyslipidemia, and blood-pressure regulation, the association may reflect broader cardiometabolic-stress regulation rather than a liability to a specific psychiatric disorder.

PRS-lipo was only associated with morning chronotype (irrespective of covariates). Lipodystrophy-related liability reflects impaired adipose storage with ectopic lipid deposition and insulin resistance rather than generalized adipose expansion (27,58). This distinction may help explain why PRS-lipo did not show the broader affective pattern observed for PRS-bfat: impaired lipid storage may have different systemic consequences from the inflammatory, adipokine, and neuroendocrine disturbances associated with excess or dysfunctional adipose tissue (50,58). Adipose tissue clocks also regulate daily variation in lipid storage, lipolysis, and insulin sensitivity, providing a plausible biological connection with sleep timing and circadian preference. The persistent association with morning chronotype should not, however, be interpreted as a beneficial consequence of lipodystrophy-related liability but may reflect shared genetic influences on lipid handling and circadian regulation. In the joint models, PRS-obes, PRS-lipo, and PRS-resgly each remained associated with morning chronotype, supporting contributions from several components of T2D liability rather than a single score.

No PRS-liver association survived FDR correction. The small number of contributing variants may limit power, so this should not be interpreted as evidence that hepatic metabolism is unrelated to psychiatric or sleep phenotypes.

### Limited Evidence for BD and Suicidality

No T2D cluster-specific PRS was associated with BD or suicidality after FDR correction. For BD, this contrasts with evidence of shared genetic liability with obesity and T2D, including *TCF7L2*, after accounting for BMI (59,60), and preliminary metabolic intervention studies (61). These broader relationships may not map onto a single T2D pathway, and the relatively small BD sample limited the power. Similarly, although diabetes is epidemiologically associated with suicidality (62), large genetic studies predominantly implicate brain-expressed and psychiatric-risk pathways (63), with limited independent cardiometabolic PRS contributions once psychiatric risk is considered (64). Overall, the findings provide little evidence that BD or suicidality is preferentially associated with a specific T2D pathway, although smaller effects cannot be excluded.

### Methodological Considerations and Limitations

Several methodological considerations and limitations should be noted. First, although our study used large-scale UKB data, participants are generally healthier and have a higher socioeconomic status than the general population, which may limit generalizability and introduce selection bias, including additional bias related to the first MHQ participation (47,48). Phenotypes also differed in their timing and ascertainment: several dimensional and behavioral traits were assessed using brief self-report items at baseline, whereas psychiatric diagnoses were derived retrospectively from the MHQ several years later. Therefore, baseline measures may have missed subsequent onset, whereas retrospective reporting may have also introduced recall error and complicated comparisons across outcomes.

The 4 MDD-derived outcomes were based on overlapping information from the same participants. Their inclusion increases the number of tests while contributing only partly independent information; therefore, concordant associations across these outcomes should not be interpreted as independent replication. Sample sizes also varied substantially between phenotypes. The secondary White European analysis was considerably smaller and showed limited cross-sample consistency after FDR correction. Lower statistical power may partly explain this pattern but confirmation in larger independent cohorts remains important, particularly for associations supported only in the primary sample.

The analyses included participants of White British or White European ancestry, restricting generalizability to other populations. The number of variants contributing to each mechanistic cluster also differed, and 11 variants were unavailable. These differences may have reduced the variance captured by some scores and lowered power, particularly for clusters defined by relatively few variants. However, the impact is unlikely to be uniform because the omitted variants may differ in effect size and biological contribution across clusters. Furthermore, although pairwise correlations between the PRS were small, the clusters represent predominant biological patterns rather than entirely discrete or mutually exclusive mechanisms.

A further consideration is that UKB contributed to the Suzuki *et al.* (27) discovery GWAS. Residual overlap between the discovery and target samples would be expected to inflate PRS associations through overfitting rather than attenuate them (65). The extent of overlap could not be quantified, but even modest inflation may be relevant given the generally small effect estimates. The interpretation of the sequential models also depends on the covariate considered: deprivation and smoking may primarily capture potential confounding, whereas BMI may lie on the pathway linking obesity-mediated genetic liability to psychiatric outcomes, and medication use may be both a consequence of psychiatric illness and a contributor to metabolic health. The models, therefore, provide complementary views of the associations but cannot distinguish confounding from mediation or establish causal direction.

### Implications and Future Directions

Our findings provide evidence that psychiatric phenotypes align differentially with T2D mechanisms, providing hypotheses for independent replication and more targeted causal investigation rather than immediate clinical prediction. Future studies should test these associations in larger, independent, and ancestrally diverse cohorts. Longitudinal and causal analyses are needed to distinguish shared genetic architecture, mediation through metabolic traits, and directional effects.

### Conclusions

Associations between T2D genetic pathways and psychiatric phenotypes were heterogeneous. The strongest evidence involved adiposity-related liability, particularly atypical MDD, whereas other pathways showed more selective relationships with affective traits, chronic pain, sleep duration, and chronotype. These findings support a pathway- and phenotype-specific framework for investigating shared metabolic and psychiatric biology.

## Supplementary Material

Supplemental Tables S1–S19

Key Resources Table
